# Osteoarthritis: a narrative review of molecular approaches to disease management

**DOI:** 10.1186/s13075-023-03006-w

**Published:** 2023-02-18

**Authors:** Loay A. Salman, Ghalib Ahmed, Stephanie G. Dakin, Benjamin Kendrick, Andrew Price

**Affiliations:** 1 Present Address: Nuffield Department of Orthopaedics, Rheumatology and Musculoskeletal Sciences, Botnar Research Centre, University of Oxford, Windmill Road, Oxford, OX3 7LD UK; 2 Orthopedics Department, Hamad General Hospital, Hamad Medical Corporation, PO Box 3050, Doha, Qatar

**Keywords:** Osteoarthritis, Posttraumatic, Animal models, Treatment, Pharmacological, Biomechanics

## Abstract

Osteoarthritis (OA) is a chronic, progressive degenerative whole joint disease that affects the articular cartilage, subchondral bone, ligaments, capsule, and synovium. While it is still believed to be a mechanically driven disease, the role of underlying co-existing inflammatory processes and mediators in the onset of OA and its progression is now more appreciated. Post-traumatic osteoarthritis (PTOA) is a subtype of OA that occurs secondary to traumatic joint insults and is widely used in pre-clinical models to help understand OA in general. There is an urgent need to develop new treatments as the global burden is considerable and expanding. In this review, we focus on the recent pharmacological advances in the treatment of OA and summarize the most significant promising agents based on their molecular effects. Those are classified here into broad categories: anti-inflammatory, modulation of the activity of matrix metalloproteases, anabolic, and unconventional pleiotropic agents. We provide a comprehensive analysis of the pharmacological advances in each of these areas and highlight future insights and directions in the OA field.

## Introduction

Osteoarthritis (OA) is a chronic, progressive degenerative whole joint disease that affects the articular cartilage, subchondral bone, ligaments, capsule, and the synovium [1]. OA was earlier considered as a wear and tear mechanical disease that causes cartilage degeneration; however, it is now understood that the cross-talk between various joint structures and local inflammation is a central aspect of the underlying pathophysiology [2].

The stratification of OA into various phenotypes is becoming widely accepted. Post-traumatic OA (PTOA) is a subtype of OA that occurs secondary to traumatic joint insults such as fractures or injury to the soft tissues, such as chondral surfaces, ligaments, tendons, and menisci or even surgical intervention to the joint [3–6]. PTOA accounts for approximately 12% of all cases of symptomatic OA [7]. While it can potentially affect any injured joint, it is most prevalent in the ankle and knee [3, 7], PTOA accounts for up to 78%, 10%, 8%, and 2% of all ankle, knee, shoulder, and hip OA cases, respectively [3, 7–9].

PTOA shares many clinical, radiological, and genetic similarities with non-traumatic OA [10]. What differentiates PTOA is that it has a clear starting point, providing an excellent opportunity for intervention and treatment as early as the time of injury [10–13]. Therefore, post-injury laboratory and animal models have been widely adopted to investigate the association between injury and OA and help exploit the intracellular processes seen in these same injured tissues to advance our understanding of OA pathways as a whole. Several injury induced-models have been utilized to study OA including surgical transection models of the meniscus or anterior cruciate ligament (ACL), controlled external loading such as ACL rupture (ACLr), or destabilization of the medial meniscus (DMM) models [10, 14, 15].

Over the past 20 years, remarkable progress has been made in osteoarthritis research; however, many questions remain unanswered due to the complexity of OA pathophysiology. It is still believed to be a mechanically driven disease; however, the role of the underlying co-existing inflammatory processes and mediators in the onset of OA and its progression is now more appreciated [10]. A complete understanding of the pathophysiology of OA would enable identification of potential therapeutic targets.

Numerous therapeutic agents have been suggested for OA [16–18]; however, there is still no definitive treatment. This review will focus on the recent pharmacological advances in the treatment of OA and summarize the most promising therapeutic agents (Table 1), based on their molecular effects, which are broadly classified into anti-inflammatory, modulators of the matrix metalloproteases activity, anabolic, and unconventional pleiotropic agents. We will highlight the complex pathophysiology of OA with an overview of the biomechanics, inflammation, and other OA associated factors. Finally, we will discuss the evolving concepts and future directions in this field.

**Table 1:** A comprehensive summary of the most promising OA pharmacological agents

Agents	Molecular target(s)	Effect
Anikanra	IL-Ra	Anti-inflammatory
IA dexamethasone	IL-1b, IL-6, and IL-8, MMPs	Anti-inflammatory Chondroprotection
Sivelestat sodium hydrate	TNF-α and IL-6 through NF-kB and HMGB1.	Anti-inflammatory Chondroprotection
JQ1 and flavopiridol	-Brd4 and CDK9 -IL-1, IL-6	Chondroprotection Reduce Synovitis
Antioxidants: NAC, Mn 3 porphyrin, vitamin E and C	ROS	Chondroprotection Prevent apoptosis
Caspase inhibitors	Caspases	Chondroprotection Prevent apoptosis
SS-31 (mitoprotective peptide)	Mitochondrial membrane, cardiolipin.	Maintain MT function and structure Chondroprotection
Doxycycline	MMP-13	Chondroprotection Reduce synovitis
EMBC injection	MMPs	Chondroprotection Osteophyte formation inhibition
Ipriflavone	IHH pathway MMP-13 Collagen X	Chondroprotection
SOST/sclerostin	WNT signaling NB-kB MMP-2 and MMP-3	Chondroprotection Osteophyte formation inhibition
AMD3100	PI3K/AKT pathways MMP-1, MMP-3, MMP-13	Chondroprotection
Remifentanil	PI3K/AKT pathways	Chondroprotection
Bisphosphonates: ALN, zoledronic acid	Bone turnover Osteoclastic activity	Regenerative Remodeling Chondroprotective
GF: BMB7 Sprifermin (FGF18)	Chondrocyte repair	Regenerative Chondroprotection
MSCs	Chondrocyte repair Inhibits: IL-6, IL-8, CCs, CXCs, cell adhesions (ICAMS-1, VCAMS-1). Stimulates: IL-4, IL-10 Phagocytic PMNs M2 macrophages	Regenerative Immunomodulator Anti-inflammatory
NBQX	AMPA/KAINATE glutamate receptor	Anti-inflammatory Chondroprotection Reduce pain
IA liposomal adenosine	A2A receptor	Chondroprotection
IA bortezomib	Proteasome inhibitor	Ameliorate synovial lymphatic drainage Chondroprotection
Erlotinib	Integrin a1b1 protection via reduction of EGFR signaling	Chondroprotection Bone volume preservation
KUS121 (VCP modulator)	TNF-α and IL-6 MMP-1, MMP-13, and ADAMTS5	Chondroprotection Anti-inflammatory Prevent apoptosis
Rebamipide	NF-kB, increased COL2A, TIMP3, TGFb, and FGF2 Decreased IL-1b, TNF-α, NF-kB, MMP-3, MMP-13, and ADAMTS5	Chondroprotection Anti-inflammatory Improved Histology
Amino sugars: N-acetylglucosamine	Chondrocytes Cytokines	Anti-inflammatory
Lubricants: HA, lubricin	Synovial lubricant deficiency	Lubrication Chondroprotection

## Method

A thorough literature review was performed using PubMed/MIDLINE, Web of Science, and Google Scholars databases and searched from inception till June 2022 with the following search terms: “Pathophysiology,” “Epidemiology,” “Inflammation”, “Biomechanics,” “Treatment,” “Therapy,” “Pharmacological,” “Intervention,” and “Osteoarthritis.” This yielded a total of 560 articles which were screened based on title/abstract to identify original research work and review articles written in English within the past 10 years. No restrictions were placed on the types of study design. Inclusion was limited to relevant references, mainly related to the pharmacological treatment of OA. Articles focusing on other perspectives of OA and inaccessible full texts were excluded. We also included several references not identified by the search criteria which were known to the author or were manually selected from the reference lists contained within the screened articles. Selected references were then reviewed and finalized by two authors independently. As a result, 66 articles met the eligibility criteria and were included in this review. Additionally, this narrative review was conducted in line with the Scale for the Assessment of Narrative Review Articles (SANRA) quality assessment tool [19].

## Results

### Pathophysiology: biomechanics and inflammation

**Fig. 1:**
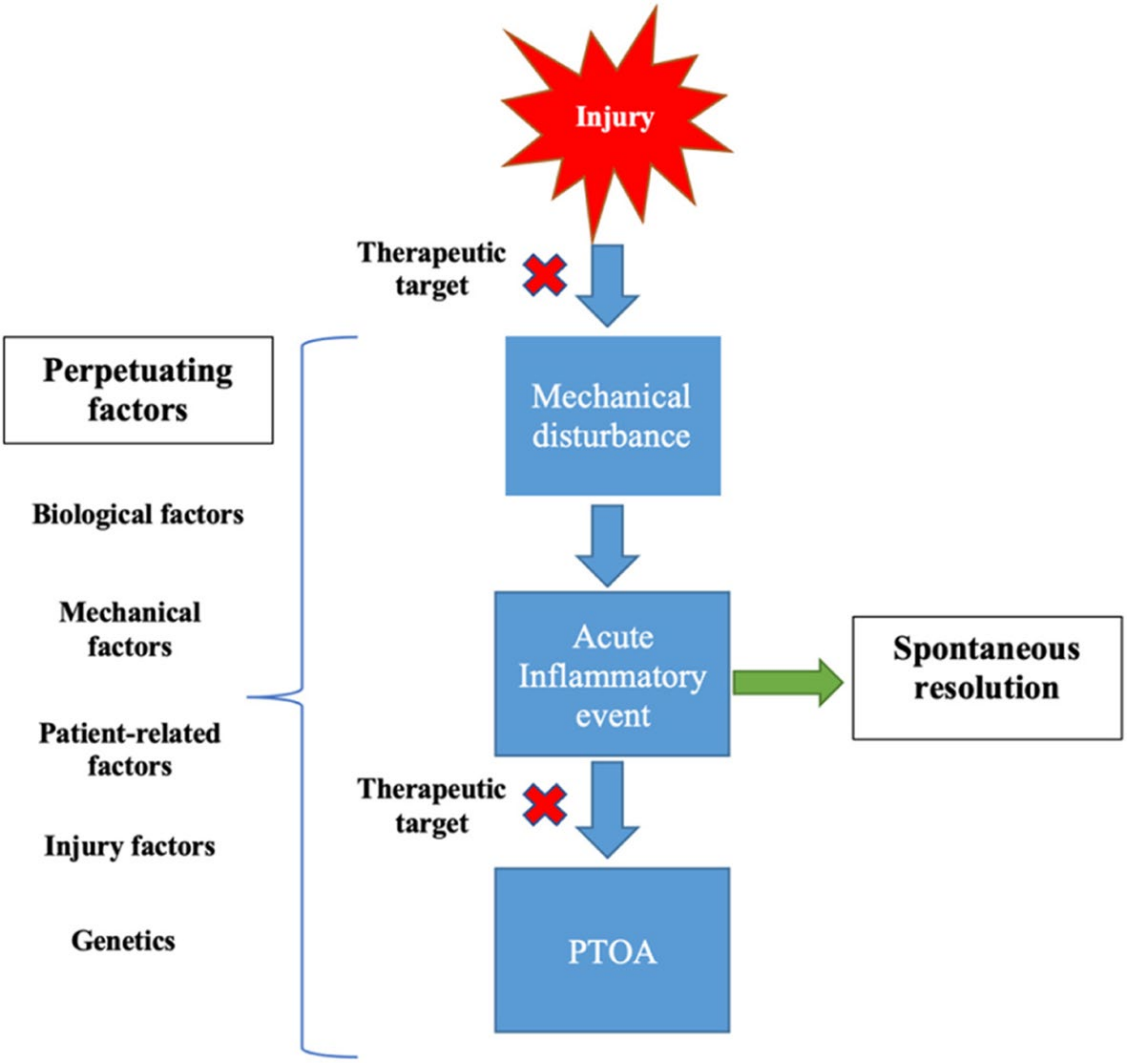
PTOA pathogenesis. Risk factors aggravating the process. Potential therapeutic targeting points are pointed out with red X

PTOA pathogenesis occurs from the point of injury to the time of presentation of OA symptoms (Fig. 1). Following a traumatic injury, a state of mechanical imbalance and overload occurs, which triggers several inflammatory signaling pathways, such as the nuclear factor kappa B (NF-kB), cyclooxygenase-2 (COX-2), inducible nitric oxide synthase (iNOS), and poly adenosine diphosphate (ADP)-ribose pathways in the synoviocytes [13, 20]. The activation of these inflammatory cascades along with the continuous mechanical disturbance increases the levels of inflammatory mediators and other matrix destructive enzymes, resulting in chondrocyte apoptosis, matrix degradation, leukocyte recruitment, and other structural and molecular changes associated with OA [10, 11]. The acute inflammatory phase either progresses to OA or resolves spontaneously, depending on the presence of aggravating factors. The risk factors that stimulate the disease’s progression are similar to that described for OA [10] (Fig. 1).

A complete understanding of the fundamental biological pathways and mediators involved in OA (Table 2) would enable creating target-based, effective therapies. Pharmacological interventions have been vigorously investigated [17]; however, they are yet to be applied clinically. The current strategies in OA management is mainly conservative, with analgesics and physiotherapy in the early stages and reconstructive or replacement surgeries at advanced stages [13]. Anatomical reconstructive procedures have advanced in terms of techniques and improved outcomes; however, evidence supporting their role in preventing OA is still insufficient [13]. Anterior cruciate ligament (ACL) injury renders the knee joint mechanically unstable and expedites osteoarthritic changes [18]. It was believed that ACL reconstruction restored the joint’s stability and prevented the development of OA; however, a large meta-analysis of 38 studies demonstrated that OA occurs even after ACL reconstruction and restoration of joint stability [21]. Therefore, apart from mechanical factors, it is critical to address the molecular pathways involved in the development of OA. This necessitates an early and robust intervention, through two possible strategies: Early, preventive interventions that target the disease process at the onset, or approaches that modify the disease prognosis (Fig. 1).

**Table 2:** Acute inflammatory phase mediators involved in OA

Cytokines: IL-1, IL-6, IL-8, 1L-17, TNF-α	
Metalloproteinases: MMP-1, MMP-3, MMP-8, MMP-9, MMP-13, ADAM-TS5	
Reactive oxygen species (ROS)	
Caspases	
Inflammatory cells (M1-macrophages, lymphocytes)	
Cartilage degradation products (proteoglycan, collagen)	
Innate immune cells	

### Anti-inflammatory agents for the treatment of OA

#### Cytokines

The pathogenic role of proinflammatory mediators, such as cytokines and chemokines, is well understood [11, 12]. Various cytokines, such as IL-1, IL-6, IL-17, and TNF-α, are involved in the acute inflammatory phase post-joint injury, with prominent crosstalk occurring between the articular cartilage and the synovium. Therefore, inhibition of these cytokines is a promising therapeutic strategy. An imbalance between the levels of pro-inflammatory (high levels of IL-1, IL6, and IL8) and anti-inflammatory (low levels of IL-1Ra, IL-4, and IL-10) cytokines is characteristic of the acute inflammatory phase [11, 12]. The most promising cytokine targeting therapy is the inhibition of IL-1 using IL-1Ra, currently in clinical trials [17, 22]. Inhibition of IL-1 was therapeutically effective in alleviating the progression of OA in animal models [23]. Early intervention with a single, small intraarticular dose of the human recombinant IL-1Ra, Anakinra, significantly alleviated the arthritic changes, reducing articular degeneration and synovitis in C57BL/6 mice with tibial plateau articular fracture [24]. The continuous systemic administration of IL-1Ra, however, yielded no therapeutic effect and, interestingly, led to a greater joint deterioration. IL-1Ra was proved safe in a multicenter randomized clinical trial (RCT) in patients with knee osteoarthritis [25]. IL-Ra (a single 150 mg dose) substantially improved the functional knee outcome measures, with reduced knee pain at 2 weeks, in a pilot trial for the treatment of acute (less than a month since injury) ACL injuries [22].

The inhibition of other proinflammatory cytokines, such as IL-6, IL-17, and TNF-α, is expected to reduce degenerative cartilage changes, synovial inflammation, and lubrication problems [13]; however, inhibiting TNF-α is not therapeutically effective in PTOA [23, 24].

In a rabbit PTOA model, intraarticular administration of dexamethasone immediately after surgical drill injury attenuated proinflammatory (IL-1β, IL-6, and IL-8) cytokines and OA-like histological changes [26]. Glucocorticoids exhibit anti-inflammatory effects in different tissues through the suppression of prostaglandins [27], inflammatory cytokines [28], nitric oxide [29], and other oxygen-derived radicals [30], making them an attractive therapeutic choice [31]. Low dose of dexamethasone offers significant chondroprotection, by reducing the loss of extracellular matrix (ECM) proteoglycans and collagen in an IL-1 rich environment and by reducing the loss of glycosaminoglycans (GAGs) even in the presence of inflammatory mediators, such as TNF-α, in an in vitro study in human chondrocytes [31].

Sivelestat sodium hydrate ameliorated knee PTOA in a rat model, acting via NF-kB and HMGB1; therefore, it could be potential treatment option for OA [32]. The expression of both of these factors is suppressed, indicating a potential anti-inflammatory response. The production of the pro-inflammatory cytokines, TNF-α and IL-6, is also significantly reduced. Moreover, after receiving a once-weekly dose of 10 mg/kg for four consecutive weeks, there is a dramatic reduction in cartilage degeneration.

JQ1 and flavopiridol suppressed the development of OA in vitro and in an in vivo mouse model of ACL rupture (ACLr) [33]. This was achieved via inhibiting the rate limiting enzymes of the primary response genes (PRGs), namely, bromodomain-containing-protein-4 (Brd4) and cyclin-dependent-kinase-9 (CDK9). In cartilage explants, they work synergistically in preventing the activation and release of IL-1β-induced inflammatory factors and glycosaminoglycan. In vivo treatment with JQ1 and flavopiridol causes a significant suppression of IL-1 and IL-6 expression, MMPs, synovial inflammation, and other joint-associated inflammatory pathways, such as iNOS and COX2.

#### Mitochondria-associated pathways

**Fig. 2:**
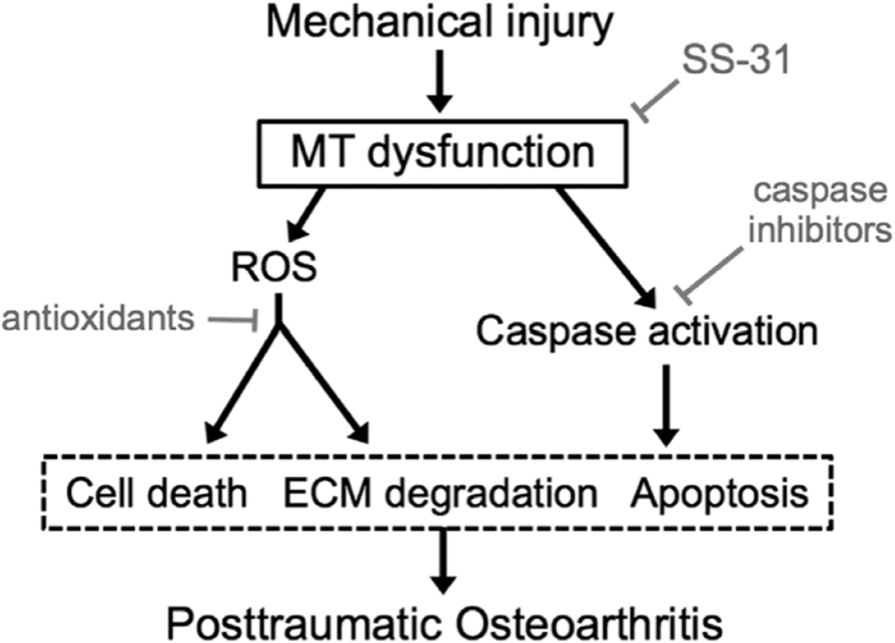
Effect of mechanical injury on mitochondria-associated pathways. Effects on MT dysfunction, oxidative response, and caspase activation leading to cell death, ECM degradation, and apoptosis and subsequently PTOA. Potential inhibitory roles of certain pharmacological interventions are depicted. Adapted from Delco et al. [15]. MT, mitochondria; ROS, reactive oxygen species; ECM, extracellular matrix

Disruption of mitochondrial structure and/or function is one of the earliest pathogenic mechanisms that trigger the onset of OA and its progression [34]. In the sub-acute phase following injury, chondrocyte apoptosis and articular degeneration are facilitated by mitochondrial damage, resulting in decreased respiratory function and proteoglycan content and an imbalance between the anabolic and catabolic pathways in the ECM, particularly the expression of MMP-13, as observed in a mouse DMM model [35, 36]. The pathways downstream of the mitochondrial pathways (Fig. 2), such as the electron transport chain and Bax/Bak pathways, are activated, resulting in the release of oxygen radicals and caspases, respectively. Antioxidants and caspase inhibitors are used to counteract these effects [17, 36]. The antioxidants, such as N-acetyl cysteine, Mn 3 porphyrin (a superoxide dismutase mimetic), and vitamins E and C, exhibit promising chondroprotective effects in animals and in ex vivo human studies. They attenuate both mechanically induced apoptosis and the expression of ECM degrading enzymes [16, 37]. Caspase inhibitors prevented chondrocyte apoptosis in preclinical studies [17]; however, their clinical efficacy in humans is not proven. The mitoprotective peptide, SS-31, protects an important phospholipid constituent of the mitochondrial inner cell membrane, cardiolipin [36], thereby maintaining the integrity of the electron transport chain and ensuring proper ATP production, reduced ROS production, and reduced mitochondrial-induced cell death. The therapeutic efficacy of SS-31 was established in an ex vivo model of PTOA [36]. SS-31 prevents trauma-induced chondrocyte apoptosis, cell membrane damage, cartilage GAG loss, and matrix degeneration. Although inherent challenges with targeting mitochondrial-associated pathways exist as effects are not tissue-specific, the safety of SS-31 in humans has been reported [38], enhancing its potential as a candidate for OA therapy.

### Inhibitors of the action of matrix degrading enzymes

#### Doxycycline

Doxycycline is a broad-spectrum tetracycline antibiotic and inhibited the progression of joint OA in a murine ACLr model [38]. There is a positive correlation between doxycycline concentrations and the degree of MMP-13 inhibition, as observed by immunohistochemistry. There is a marked reduction in MMP-13 levels and significantly less cartilage damage and synovial inflammation. A systematic review of seven animal studies indicated mixed results, with some positive effects of doxycycline in PTOA treatment [39], making it a promising therapeutic candidate for OA.

#### Injection of ECM blood composites

Collagen type I is one of the main components of extracellular matrix-blood composite (EMBC). It is a competitive inhibitor of MMPs, preserving the cartilage matrix. Intra-articular injection of EMBC yielded chondroprotective effects in PTOA rat models, resulting in reduced cartilage degeneration and osteophyte formation [40].

#### Ipriflavone

Ipriflavone is a dietary supplement with anabolic effects on the bone and an inhibitor of the Indian hedgehog (IHH) pathway. Stimulation of the IHH pathway is crucial in the progression of OA, resulting in degenerative changes through the upregulation of MMP-13 [41, 42]. Ipriflavone mitigates cartilage degeneration in vivo (rats) and in vitro (human cartilage explants), by reducing the levels of MMP-13 and collagen type X.

#### Sclerostin

Sclerostin (SOST) is a Wnt antagonist that inhibits bone osteoblastic activity. The protective role of sclerostin was studied in a tibial compression overload model in SOST transgenic and knockout mice [43]. Prolonged sclerostin exposure resulted in the activation of the NF-kB pathway and downregulation of cartilage matrix degradation enzymes (MMP-2 and MMP-3). Sclerostin-treated mice exhibited milder OA articular changes and reduced development of osteophytes. Similar effects were observed with the intraarticular administration of recombinant sclerostin protein.

### Anabolic mediators

#### Bisphosphonates

The use of bisphosphonates is promising for OA, because of their significant bone remodeling potential and anti-osteoclastic activity. The chondroprotective effects of alendronate and its ability to preserve subchondral bone in PTOA models have been established in preclinical studies [44–47]. In a rat model of PTOA, alendronate significantly inhibited osteophyte formation by up to 51% at 8 weeks post-surgery [46] and reduced cartilage degeneration. The effects of alendronate were dose-dependent but not long-lasting in a mouse ACLr model [47]. One RCT showed that bisphosphonate zoledronic acid [48] provided better symptomatic pain relief and reduced primary knee OA structural changes when compared to placebo. A statistically and clinically significant reduction (39% vs 18%, *p* = 0.044) in knee bone marrow lesion size and numbers at 6 and 12 months was seen. Further clinical studies, on the dose, time of administration, and safety in patients with OA, are required.

#### Growth factors

##### Bone morphogenetic protein 7 (BMP-7)

BMP-7, also known as osteogenic protein-1 (OP-1), is a potent member of the TGF-b family and promoter of osteoblast differentiation. It modulates chondrocyte metabolism and protein synthesis [16]. The cartilage regenerative capacity of BMP-7 has been demonstrated in preclinical studies, making it a robust anabolic candidate for treating both OA and PTOA [16, 49, 50]. Several clinical trials of BMP-7 have been conducted in patients with knee OA [51]. Intraarticular knee administration of BMP-7 results in a toxicology profile comparable to that of the placebo group, establishing its safety; however, it could not significantly alleviate the pain [52].

##### Sprifermin (FGF18)

Sprifermin is a synthetic recombinant human FGF18 with promising anabolic implications in OA. A 5-year, multicenter RCT studied the effect of sprifermin on femorotibial joint cartilage thickness in 549 patients with symptomatic knee OA [53]. Intraarticular injections of 100 μg of sprifermin every 6 or 12 months resulted in a statistically significant improvement in total femorotibial joint cartilage thickness after 2 years. The functional outcome scores, however, were not different between the treatment and placebo groups, suggesting clinical irrelevance. Evaluation of its application in OA and further investigations on the clinical outcomes and their duration is therefore necessary.

##### Mesenchymal stromal cells (MSCs)

MSCs are multipotent heterogenous cells that differentiate into chondrocytes and, therefore, play a critical role in cartilage repair [54]. They also exhibit anti-inflammatory and immunomodulatory effects [55]. They regulate the levels of IL-1β, TNF-α, and IFN-γ, and their immunosuppressive and anti-inflammatory effects are promising for clinical applications. The exact relationship is not fully understood; however, the stimulation of anti-inflammatory cytokines, phagocytic cells, and regulatory M2 macrophages have been proposed. Intraarticular administration of MSCs effectively prevents the development of OA and preserves bone thickness, in various strains of mice [24]. Intraarticular administration of MSCs exhibits positive clinical and radiological outcomes in cartilage quality, when compared to the hyaluronan control group, in an RCT [56]. Further understanding of the specific mechanisms, tissue source, immunogenicity (allogeneic vs autogenic), storage techniques, and the doses and safety of MSC treatments in OA is required.

### Unconventional targets

#### NBQX

Glutamate and GluR are upregulated following joint injury, facilitating the onset of OA. Intraarticular administration of NBQX, a glutamate receptor inhibitor, in an ACLr mouse model at the time of injury, suppressed inflammation, pain, and joint degeneration [57]. NBQX functions through the AMPA/kainate glutamate receptor and is more efficient than the conventional treatment using hyaluronic acid and steroids. GluR antagonists are used for treating numerous CNS conditions, establishing their safety profile in humans [57]. This makes it feasible to advance them into human trials for treating OA.

#### Intraarticular adenosine

Intraarticular adenosine is another unconventional agent for treating OA. It is an agonist of the A2A receptor and exhibits apparent chondroprotective effects. Extracellular adenosine is critical for articular cartilage homeostasis [58, 59]. Stimulation of the A2A receptor has protective effects on cartilage, and it downregulates the catabolic matrix-degrading enzymes. In addition, it increases the nuclear P-SMAD2/3/P-SMAD1/5/8 ratio, thereby shifting the chondrocyte balance to a healthier quiescent state [60].

#### Bortezomib

Bortezomib is a proteasome inhibitor that suppresses TGF-induced collagen II degradation and MMP-13 expression, in human chondrocytes [61]. The relationship between the synovial lymphatic system and the development of OA remains unclear [62]; however, it is believed that the obstruction of the joint lymphatic system exacerbates the inflammatory phase and the progression of OA. Intraarticular administration of bortezomib ameliorates synovial lymphatic drainage, cartilage loss, reduces the number of M1-macrophages, and inhibits the expression of proinflammatory genes [63].

#### Erlotinib

Erlotinib, an inhibitor of epidermal growth factor receptor (EGFR), which reduces OA-induced cartilage loss, improved subchondral bone thickness and volume owing to the protective role of integrin α1β1 and the reduction in EGFR signaling in various strains of model mice [64]. Interestingly, these effects were gender specific and observed only in female mice.

#### KUS121

KUS121, a valosin-containing protein (VCP) modulator, was effective in vitro and in a rat model of PTOA [65]. KUS121 significantly reduced the levels of the pro-inflammatory cytokines, TNF-α and IL-6, as well as the ECM catabolic enzymes, MMP-1, MMP-13, and ADAMTS5, in human articular chondrocytes. In addition, it alleviated cartilage damage and chondrocyte apoptosis in a rat model of PTOA induced by cyclic compressive load and, therefore, is a promising therapeutic option for OA.

#### Rebamipide

Rebamipide has protective effects on articular cartilage degeneration, both in vivo and in vitro [66]. A once-weekly injection of rebamipide into the knee joints of mice and the treatment of human chondrocyte explants with rebamipide increased the expression of cellular protective factors, such as COL2A, TIMP3, TGFβ, and FGF2, in chondrocytes and suppressed the expression of pro-inflammatory and catabolic factors, such as IL-1β, TNF-α, NF-κB, MMP-3, MMP-13, and ADAMTS5.

## Discussion

### Future directions

PTOA is one of the most debilitating subtypes of OA, because it affects the younger active population, resulting in a considerable impact on the healthcare system. However, it offers a massive opportunity for advancing our knowledge on osteoarthritis, understanding the underlying pathogenic mechanisms, and exploring therapeutic options. This opportunity arises from the fact that PTOA, unlike other OA phenotypes, is associated clearly with an onset event, the joint injury. Most of the studies described in this review are preclinical, conducted on animal and in vitro human chondrocytes models; however, therapeutic agents, such as IL-1Ra, dexamethasone, bisphosphonates, and MSCs are under clinical trials, with promising findings. The translation of these findings to clinical practice is challenging, because of the vast differences between lab models and humans, with respect to biomechanics, genetics, and systemic body response. Identification and validation of more sensitive biomarkers and radiographic signs with high OA predictive value will improve the practical application of the results from future clinical trials and circumvent the long-term follow-up periods. Finally, with osteoarthritis stratification gaining much recognition, precision-medicine can play key diagnostic and therapeutic roles in the field of OA, with opportunities for further exploration.

## Conclusion

The burden of OA and the lack of consensus in early treatment options was the motivation for this review. A successful pharmacological treatment, along with conservative measures, could alleviate the need for surgical interventions in managing OA. Therapeutic agents, such as IL-1Ra, dexamethasone, bisphosphonates, and MSCs are in clinical trials, with promising findings. The future direction of OA treatment includes translating experimental findings to clinical practice by designing feasible clinical trials with short-term, objective outcomes, in addition to exploring other therapeutic options, such as genetics and nanotherapy-based interventions.

## Abbreviations

OA: Osteoarthritis
PTOA: Post-traumatic osteoarthritis
ACL: Anterior cruciate ligament
ACLr: Anterior cruciate ligament rupture
DMM: Destabilization of the medial meniscus
SANRA: Scale for the Assessment of Narrative Review Articles quality assessment tool
ECM: Extracellular matrix
GAGs: Glycosaminoglycans
IL: Interleukin
MMP: Matrix metalloproteinase
TNF: Tumor necrosis factor
TGF: Transforming growth factor
NF-kB: Nuclear factor kappa B
COX-2: Cyclooxygenase-2
iNOS: Inducible nitric oxide synthase
IHH: Indian hedgehog
SOST: Sclerostin
BMP-7: Bone morphogenetic protein 7
MSCs: Mesenchymal stem cells
PRGs: Primary response genes
Brd4: Bromodomain-containing-protein-4
CDK9: Cyclin-dependent-kinase-9
